# Determination of Mutational Timing of Colistin-Resistance Genes through *Klebsiella pneumoniae* Evolution

**DOI:** 10.3390/pharmaceutics15010270

**Published:** 2023-01-12

**Authors:** Jenna M. Kuhn, Yuanpu Peter Di

**Affiliations:** Department of Environmental and Occupational Health, School of Public Health, University of Pittsburgh, Pittsburgh, PA 15261, USA

**Keywords:** *Klebsiella pneumoniae*, colistin, antimicrobial resistance, mutation timing, evolution

## Abstract

The emergence and dissemination of carbapenem-resistant *Klebsiella pneumoniae* (KP), one of the carbapenem-resistant *Enterobacteriaceae* (CRE), is now an emerging cause of antibiotic-resistant nosocomial infections associated with high rates of morbidity and mortality. Colistin, or polymyxin E, is a last-resort peptide antibiotic used to treat multidrug-resistant (MDR) Gram-negative bacterial infections including KP. Unfortunately, resistance to colistin is rising with increasing use in the clinical setting. Although clinical evidence links certain mutations to colistin resistance (COL-R) in KP, the origination and association of the mutations remain unclear. We hypothesize that the timing of COL-R mutations influences the development and progression of KP resistance to colistin. We performed planktonic and biofilm in vitro experimental evolutions of KP strain ATCC 43816 under increasing colistin concentrations to characterize the temporal regulation of critical COL-R mutations throughout COL-R progression. The resistance generation and mutation profiles of independently evolved bacterial populations with different lifestyles were compared. Genes with various functions theorize the timeline in which key mutations are generated and their roles in the progression of COL-R. Our results aim to advance the research and development of effective therapeutics to treat MDR bacterial infection as the dissemination of CRE continues to be a severe public health threat.

## 1. Introduction

The emergence of carbapenem-resistant Enterobacterales (CRE) has become a global public health threat with high mortality rates of infection and limited available antimicrobial treatment options [1]. According to the US Centers for Disease Control and Prevention 2019 Antibiotic Resistance Threats Report, CRE infections led to approximately 13,100 hospital incidents and 1100 infection-related deaths, with average case numbers remaining steady between 2012 and 2017 [2]. Additionally, the worldwide dissemination of extended-spectrum β-lactamases (ESBLs) in species of *Enterobacteriaceae* and *P. aeruginosa* has facilitated resistance to a number of β-lactam antibiotics, including carbapenems, cephalosporins, monobactams, and penicillins, as well as recent detection of resistance to aztreonam and oxyimino-cephalosporins [3,4]. A pathogen of urgent concern of the *Enterobacteriaceae* family, *Klebsiella pneumoniae* (KP), is a one of six nosocomial ESKAPE pathogens (*Enterococcus faecium*, *Staphylococcus aureus*, *Klebsiella pneumoniae*, *Acinetobacter baumannii*, *Pseudomonas aeruginosa*, and *Enterobacter* species), noted for its virulence and high potential for multidrug resistance (MDR) [5]. KP is characterized as an encapsulated, Gram-negative, nonmotile, facultative anaerobic pathogen acquired in community or healthcare settings that may cause pneumonia, urinary tract infection, soft-tissue infection, bacteremia, and meningitis, especially in immunocompromised individuals [6]. The recent emergence and rising prevalence of carbapenem-resistant hypervirulent KP (CR-hvKP) has several concerning clinical impacts due to high resistance and pathogenicity, high mortality, production of multiple carbapenemases, and gut colonization, facilitating further resistance dissemination [7]. Due to rising numbers of identified ESBLs and the global spread of CR-hvKP, there are limited treatment options available.

**Figure 1:**
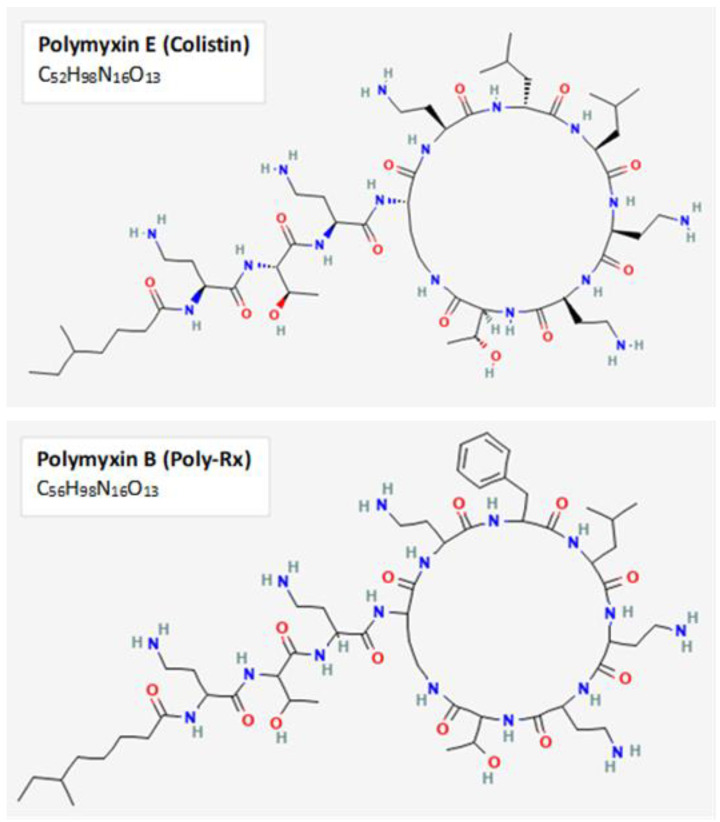
Chemical structure of polymyxins B and E. The primary polymyxins used clinically to treat Gram-negative infections are the cyclic polypeptides Polymyxin B and E. Both polymyxins enact their activity through binding to negatively-charged lipopolysaccharide and disruption of outer membrane permeability. The two-dimensional chemical structures and formulas for polymyxin B and polymyxin E displayed in this figure were obtained from PubChem, with CIDs 4868 and 5311054, respectively.

Colistin, or polymyxin E, is a last-resort antibiotic reserved for treating complex CR-hvKP infections. Polymyxins are cationic cyclic polypeptides that belong to a family of antimicrobial peptides (AMPs), which are bactericidal components of the host innate immune system present in plant and animal species, as well as some bacteria and fungi. AMPs are usually amphipathic, cationic, and approximately 15–30 amino acids in length; they impose their activity at the cell membrane of bacteria [8]. The two dominant polymyxins used clinically to treat Gram-negative bacterial infections are polymyxin B and polymyxin E (Figure 1) [9,10]. AMPs challenge the development of drug resistance due to their diverse killing mechanisms and low specificity for a given target in host cells [11].

Colistin is a cationic polypeptide that exerts its activity by binding to anionic regions of lipopolysaccharide (LPS), leading to permeabilization of the cell envelope, cell leakage, and cell death [12]. Since its displacement by other antimicrobials four decades prior, colistin has shown significant activity against KP, *P. aeruginosa*, *A. baumannii*, and other Gram-negative pathogens with low initial resistance levels [13]. However, with increased use in the clinical space, rates of colistin resistance have been on the rise through several main mechanisms, including chromosomal mutations in genes responsible for disrupting the cationic charge of LPS (PhoP/PhoQ and PmrA/PmrB two-component regulatory systems) and MgrB, a transmembrane regulator of PhoP/PhoQ signaling, as well as plasmid-mediated colistin resistance (*mcr-1-10*) [14,15]. It has also been shown that increased production of capsular polysaccharide hinders interactions between colistin with the cell membrane of bacteria, facilitating resistance [14]. While the primary mechanisms of COL-R have been identified, the relationship among resistance generation timing, population frequency, and interactions of mutations facilitating the onset and progression of COL-R has not been thoroughly investigated. The demand for further development of effective antimicrobials against the growing public health threat of MDR infections continues to grow. Now, even last-resort treatments are showing high rates of resistance.

In particular, biofilm-associated infections comprise ~65% of all bacterial infections in the clinical setting and present a serious challenge to the healthcare community due to their diversity and innate defense mechanism to protect against antimicrobials [16,17]. There are several mechanisms via which biofilms may resist antimicrobial agents. The exopolysaccharide matrix of biofilm environments may hinder penetration of negatively charged antimicrobials such as aminoglycosides [17]. Furthermore, microcolonization within the biofilm leads to waste accumulation and fluctuation of pH and CO_2_ and O_2_ partial pressures, consequently disrupting antimicrobial activity [17]. Additionally, regulation of efflux pumps, expression of antimicrobial chelating enzymes, and quorum sensing are other biofilm-associated resistance mechanisms [17]. KP biofilms may lead to invasive, chronic infections in the urinary, gastrointestinal, or respiratory tracts through cell adhesion to a surface, colony formation, biofilm maturation, and cell detachment [18]. Here, we consider both planktonic and biofilm KP lifestyles in our experimental evolution methods to better understand the timing of critical resistance mutations and their likely impact on the progression of COL-R.

## 2. Materials and Methods

### 2.1. Bacteria Strain and Culture Media

*Klebsiella pneumoniae* American Type Culture Collection (ATCC) strain 43816 was first grown on cation-adjusted Mueller–Hinton Broth 2 (MHB2) agar medium (Sigma-Aldrich, St. Louis, MO, USA) overnight, and a single colony was transferred into liquid M9 medium supplemented with ½MIC level of colistin sulfate treatment (Research Products International, Mount Prospect, IL, USA). M9 minimal growth medium was designed using M9 salts (6 g/L Na_2_HPO_4_, 3 g/L KH_2_PO_4_, 0.5 g/L NaCl, and 1.0 g/L NH_4_Cl) supplemented with 0.1 mM CaCl_2_, 2 mM L-Glutamine, 2 mM MgSO_4_, and 4% glucose to reflect the essential components required by mammalian cells and biofilm formation to prevent additional nutrient selection effects [19,20].

### 2.2. Bacterial Transfer and Resistance Selection

**Figure 2:**
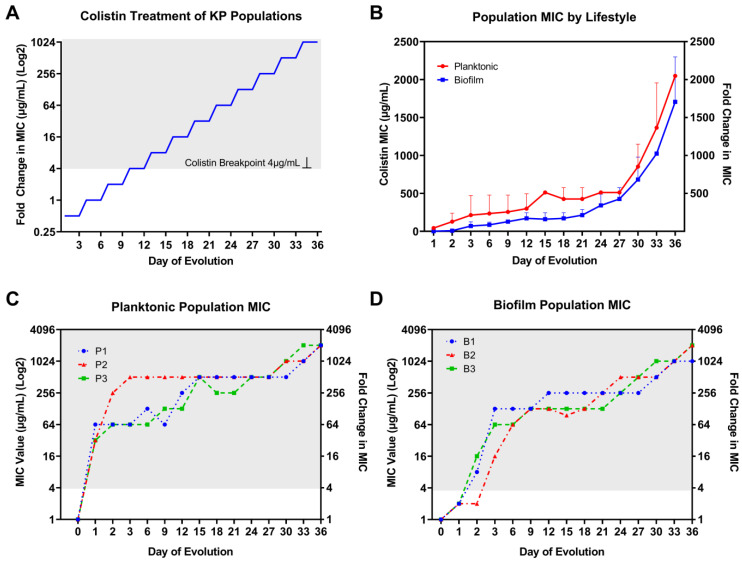
Colistin selection and population MIC result by bacterial lifestyle. (**A**) Colistin treatment for KP planktonic and biofilm populations started at ½ MIC; the selection concentrations were doubled every three days for 36 days. (**B**) Colistin population MIC results averaged among three replicate colistin-treated KP populations by bacteria lifestyle. Rapid resistance to colistin was observed for (**C**) planktonic and (**D**) biofilm individually evolved populations after 36 days of colistin selection pressure. By day 36, there was a 1024–2048-fold increase in colistin MIC compared to baseline level. The resistance is distinguished in gray background when MIC is above the CLSI-determined clinical breakpoint at 4 µg/mL for colistin.

To investigate the timing and relevance of resistance genes concerning MIC increase, we performed bacterial experimental evolution studies using a serial passage of KP planktonic culture and a single colonized biofilm bead under colistin selection pressure (Supplementary Figure S1). Three replicate populations were subjected to colistin selection according to bacteria lifestyle. For planktonic populations, 1/100 or approximately 2.5 × 10^6^ colony forming units (CFU)/mL of total overnight culture was transferred into fresh M9 media supplemented with colistin. For biofilm populations, a bead transfer-based biofilm evolution system was used according to a method described previously [21,22,23]. Briefly, a single colonized 7 mm polystyrene bead, approximately 2.5 × 10^5^ CFU/mL, was transferred into M9 medium supplemented with colistin and two sterile polystyrene beads. The sterile beads facilitate the processes of bacteria attachment, biofilm formation, and dispersal. The concentration of colistin treatment was doubled every three days to steadily enhance bacteria resistance selection. Evolution under antibiotic selection continued for 36 days without complete inhibition of growth after each doubling of treatment concentration (Figure 2A).

### 2.3. MIC Measurement

Colistin MIC was determined using the broth microdilution method established by the Clinical and Laboratory Standards Institute (CLSI) [24]. Whole-population MIC was determined for each KP population replicate at the end of every 3 days of serial passage and prior to transfer into the subsequent treatment concentration. The range of antibiotic selection began at a baseline level of ½MIC or 0.5 µg/mL for the ancestor KP clone on days 1–3 and was subsequently increased to a level of 1024 × MIC or 1024 µg/mL for the final days 33–36. Cation-adjusted Mueller–Hinton Broth 2 (MHB2) (Sigma-Aldrich, MO, USA) was used to inoculate each planktonic KP population for overnight incubation at 37 °C prior to MIC testing to increase bacterial cell count. KP biofilm populations were isolated by transferring a single colonized polystyrene bead into a 15 mL conical tube containing 2 mL of phosphate-buffered saline for ultrasonic homogenization (DPS-20 model, PRO Scientific Inc., Oxford, CT, USA). Prior to MIC testing, planktonic and homogenized biofilm populations were cultured in MHB2 medium for 24 h at 37 °C in a shaking incubator (Corning LSE 71L model, Corning, NY, USA). MIC testing was performed using sterile 96-well, microplates (Greiner, Frickenhausen, Germany). Bacterial concentration was adjusted to approximately 5 × 10^5^ CFU/mL with PBS prior to plating. MIC values were determined by measuring turbidity via optical density readings at 570 nm wavelength using a Gen 5 Microplate Reader and Imaging software (BioTek Instruments, Winooski, VT, USA, Version 3.04). Samples selected for MIC testing were preserved in 8% dimethyl sulfoxide for further genomic analysis.

### 2.4. Whole-Genome DNA Sequencing

KP (whole population or single clone) genomic DNA was extracted using a DNeasy Blood and Tissue Kit (Qiagen, Hilden, Germany). Biofilm attached to beads was dissociated by sonication in sterile PBS before DNA extraction. Planktonic cultures were centrifuged and pelleted before DNA extraction. Whole-genome DNA sequencing was performed using an Illumina NextSeq 2000 platform with a paired-end mode of 2 × 150 base pairs and sequencing depth coverage of 200 Mbp for individual clones and 650 Mbp for whole-population bacterial genomes (SeqCenter, Pittsburgh, PA, USA).

### 2.5. Comprehensive Mutation Analysis

Data preprocessing for raw sequencing data included gentle quality trimming using trimmomatic (version 0.38, parameter: PE -phred33 LEADING:20 TRAILING:20 SLIDINGWINDOW:4:20 MINLEN:70) to filter out low-quality and unpaired reads before computational mutation analysis [25]. Bowtie2 (version 2.4.1), as part of the breseq workflow, was used to build an index of the reference genome and align reads to the index reference genome *K. pneumoniae* strain ATCC 43816 (serotype O1:K2) [26,27]. The reference genome was downloaded from NCBI GenBank with accession number SRR13008124. Breseq was used to detect mutations relative to the reference genome in consensus mode and polymorphism mode for clonal and mixed-population samples, respectively [28]. Both mixed-population and clonal samples were analyzed to identify and confirm the most integral COL-R mutations generated with increased selection. Breseq gdtools COMPARE subcommand was used to create side-by-side mutation comparison tables for individual populations to show how genetic variants and their frequencies change over time.

### 2.6. Mutation Selection Criteria

A series of criteria were distinguished to select relevant genes and their corresponding mutations of interest. Mutation prediction using breseq and mutation comparison tables were designed and analyzed to identify alterations in genes that were observed in independently evolved populations at greater than 20% frequency. Most of these mutations increased in frequency with enhanced selection pressure or became fixed at 100% frequency in these populations. We studied the function of genes for which these mutations were observed to speculate their potential role in and importance for the resistance mechanism. Using a thorough review of the current literature, we noted mutations that have been previously identified as critical for colistin-resistance development. In addition, we included mutations that have not been studied extensively in *K. pneumoniae* to theorize how they may be influential in the resistance pathway according to their timing and associated gene functions.

### 2.7. String Test for Hypervirulence

The string test was performed to assess changes in hypervirulence following ½MIC colistin treatment for planktonic KP populations. Tryptic soy agar plates containing 40 mg/L Congo Red (Sigma, MO, USA) and 20 mg/L Coomassie Brilliant Blue (Bio-Rad, Watford, UK) dyes were prepared and used to plate various bacterial dilutions to achieve comparable single-colony counts between each treated population and the evolution ancestor clone. A sterile inoculation loop was used to touch the surface of each single colony and measure the length of mucoid string produced. A positive test was determined for a mucoid string of 5 mm or greater in length, a feature observed clinically to define the hypervirulent phenotype [29]. Statistical comparisons between treatment populations and the original clone were made using one-way ANOVA. A *p*-value < 0.05 was considered to be statistically significant.

## 3. Results

### 3.1. Rapid MIC Increase in Colistin-Treated KP Populations

The baseline MIC for the colistin-susceptible ancestor KP clone (ATCC 43816) was determined to be 1 μg/mL. Rapid increases in MIC were observed for individually evolved KP populations through experimental evolution under colistin selection with increasing concentrations (Figure 2A) independent of bacteria lifestyle (Figure 2B). However, there was a slower rate of MIC increase within the first 3 days and for the extent of evolution for biofilm- compared to planktonic-evolved KP. For planktonic-evolved KP populations, the most substantial jumps in MIC occurred during the first 3 days of colistin selection. More gradual and consistent twofold increases occurred every 3 days afterward and for the remaining days of evolution. Compared to the planktonic lifestyle, biofilm populations showed modest (twofold) increases from baseline MIC after 24 h of incubation at ½MIC level treatment. After 24 h of antibiotic selection, colistin MIC increased over 32-fold for planktonic populations and twofold for biofilm populations (Figure 1D and Figure 2C). By day 3, planktonic populations 1–3 showed 64-, 512-, and 64-fold increases in MIC compared to 128-, 16-, and 64-fold increases for biofilm populations 1–3. Similar to planktonic populations, after the first 3 days of selection, biofilm populations showed consistent 2–4-fold increases in MIC for the remainder of the 36 day evolution experiment. The CLSI breakpoint for colistin of 4 µg/mL was surpassed after only 24 and 48 h evolution at ½MIC level of selection for planktonic and biofilm lifestyles, respectively. After day 27, KP showed further twofold increases in MIC with no hindrance in growth under selection pressure for both lifestyles. Following 36 days of selection, 2048-fold increases in colistin MIC were observed for all three planktonic populations (Figure 2C) as well as 1024-, 2048-, and 2048-fold increases in biofilm populations 1–3, respectively (Figure 2D).

### 3.2. Temporal Regulation of COL-R Mutations in Planktonic KP

**Figure 3:**
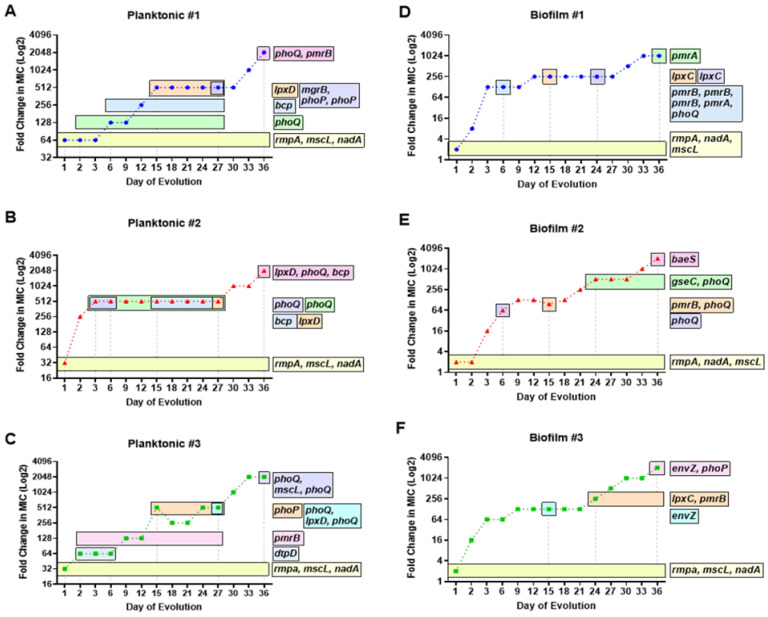
Temporal regulation of colistin-resistant mutations in planktonic and biofilm KP populations. Timelines of acquired mutations having mutation frequencies greater than 10% in known and theorized COL-R genes are represented by horizontal bars throughout a 36 day evolution. The duration of evolution in which the COL-R mutations exist within each planktonic (**A**–**C**) and biofilm (**D**–**F**) population is compared against colistin MIC. For both lifestyles, mutations in genes *rmpA*, *nadA*, and *mscL* were generated after 24 h at ½ MIC colistin selection and were subsequently fixed in each population. Planktonic populations 1 (**A**) and 2 (**B**) acquired a mutation in *bcp* around midpoint of evolution followed by mutations in *lpxD*. Biofilm populations 1 (**D**) and 3 (**F**) acquired mutations in *lpxC* around midpoint of evolution. Mutations in two component system sensor regulator genes *phoQ*/*phoP* and histidine kinase genes *pmrB*/*pmrA* appeared sporadically, in relation to notable (at least twofold) increases in colistin MIC.

After 24 h of ½MIC level colistin selection, all three replicate planktonic KP populations showed an insertion in mucoid phenotype A regulator RmpA, as well as single-nucleotide polymorphisms (SNPs) in quinolinate synthase NadA and large-conductance mechanosensitive channel protein MscL, at nearly 100% frequency (Table 1, Figure 3A–C). These mutations coincided with a substantial rise in colistin MIC equivalent to 64-, 32-, and 32-fold increases compared to baseline MIC level (1 µg/mL) for populations 1–3, respectively. By day 6 of selection, KP population 1 showed an SNP in two-component system sensor histidine kinase PhoQ at 93% frequency, which aligned with a 128-fold increase in colistin MIC compared to baseline. In addition, this mutation increased to 100% frequency in population 1 in accordance with a 512-fold increase in colistin MIC. By day 36, a SNP at a new position in *phoQ* was acquired at 100% frequency as well as an SNP in two-component system sensor histidine kinase PmrB at high frequency, in line with a 2048-fold increase in colistin MIC compared to baseline (Figure 3A). Despite detection at different positions, mutations in *phoQ* were persistently observed from day 6 onward until day 36, which may contribute to the continued enhanced colistin resistance (Table 1, Figure 3A).

For planktonic population 2, two different SNPs in *phoQ* acquired by day 3 of selection corresponded with a 512-fold increase in colistin MIC. This dramatic rise in MIC during the first 3 days of selection was prominent in population 2, compared to populations 1 and 3, which showed more gradual increases over selection time (Figure 3A–C). Population 2 showed a similar pattern of *phoQ* SNPs generated at different positions through the course of evolution to that of population 1 (Table 1). Colistin MIC level remained consistent from days 3 to 27, in alignment with the duration of a unique SNP in *phoQ* and an SNP in thioredoxin-dependent thiol peroxidase BCP, observed on days 15–27. The final jump in MIC (2048-fold) was linked to mutations at unique positions of UDP-3-O-(3-hydroxymyristoyl)glucosamine N-acyltransferase LpxD, *phoQ*, and *bcp*, all at 100% frequency. Interestingly, no *prmB* mutations were observed for population 2 for the extent of colistin resistance evolution (Table 1, Figure 3B).

Planktonic population 3 showed a unique pattern of SNPs in *phoQ* and *pmrB* with enhanced selection. On day 6, a SNP in *pmrB* was acquired and increased to 86.3% frequency by day 15. On day 15, a substantial rise in MIC was seen (512-fold) along with an additionally acquired SNP in two-component system response regulator PhoP that exists in the population until day 27 (Table 1, Figure 3C). On day 27, three unique mutations in *phoQ* (two positions) and *lpxD* were observed for which the MIC was maintained at 512-fold compared to baseline. The *phoQ* mutation seen at the same position as population 2 occurred at 73% frequency in population 3 on day 36, consistent with a 2048-fold increase in colistin MIC. The shifts in *phoQ* SNP positions with increased selection pressure likely contributed to the enhanced evolution of colistin resistance. The temporal regulation of COL-R mutations can be seen via the generation of key mutations sequentially, in alignment with considerable increases in MIC, with many alterations rising in population frequency or becoming fixed with enhanced selection pressure (Table 1, Figure 3A–C).

### 3.3. Temporal Regulation of COL-R Mutations in Biofilm KP

Similar to the “first wave” of colistin resistance acquired in planktonic KP, after 24 h selection, all three replicate KP biofilm populations showed mutations in *rmpA*, *nadA*, and *mscL* at 100% frequency (Table 2, Figure 3D–F). Insertions in *rmpA*, and SNPs in *nadA* and *mscL* coincided with 128-, 16-, and 64-fold increases in colistin MIC by day 3 of selection for biofilm populations 1–3, respectively. After 6 days of selection, biofilm population 1 acquired three SNPs in *pmrB* at unique positions, as well as an SNP in *phoQ*, consistent with a 128-fold increase in MIC. These mutations generated by day 6 were lost. Subsequent SNPs in *phoQ* and UDP-3-O-acyl-N-acetylglucosamine deacetylase LpxC were later acquired by day 24, which likely contributed to additional MIC increase (Figure 3D). In addition, a SNP in *lpxC* by day 15 aligned with another twofold increase in MIC, equivalent to a 256-fold increase from baseline. The final day of selection showed a 1024-fold increase in MIC, which overlapped with the timing of a deletion in PhoP/PhoQ regulator and DNA-binding transcriptional repressor MgrB–KdgR and a SNP in two-component system response regulator PmrA (Table 2, Figure 3D).

For biofilm population 2, a similar timeframe for generating two-component system sensor histidine kinase mutations was seen to that of population 1 (Table 1, Figure 3E). By day 6, an SNP of 75% frequency was acquired in *phoQ*, consistent with a 64-fold increase in MIC from baseline. Two unique SNPs in *pmrB* and *phoQ* were generated by day 15 (Figure 3E), with a slight twofold increase in MIC. A 512-fold increase in MIC from baseline occurred by day 24, with the generation of an SNP in *phoQ* and deletion in two-component system sensor histidine kinase QseC, which remain fixed in the population for the extent of the selection. By day 36 of selection, a newly appeared mutation in two-component system sensor histidine kinase BaeS aligned with a 2048-fold increase in colistin MIC from baseline (Table 2, Figure 3E).

For biofilm KP population 3, a delay in the generation of two-component system sensor histidine kinase mutations was observed until day 15 of selection (Table 2, Figure 3F). By day 15, a mutation in two-component system sensor histidine kinase EnvZ was generated, consistent with a 128-fold increase in MIC from baseline. Another twofold increase in MIC was seen by day 24, for which mutations in *lpxC* and *pmrB* were acquired and fixed for the extent of selection. By day 36 of selection, two unique mutations in *envZ* and *phoP* were generated, which aligned with a 2048-fold increase in MIC from baseline (Table 2, Figure 3F). Through monitoring the MIC change via increasing concentrations of colistin selection, key mutations in two-component system sensor and regulators, as well as SNPs in *lpxC*, appear to drive COL-R in biofilm populations. The modification in mutation position for these critical genes and the increase in population frequency over time are also likely contributing factors to enhanced colistin resistance, independent of bacterial lifestyle. Next, we aimed to further characterize COL-R in KP by studying the timing of these mutations in relation to gene function.

### 3.4. Timing of Colistin Resistance Mutations by Functional Roles

**Figure 4:**
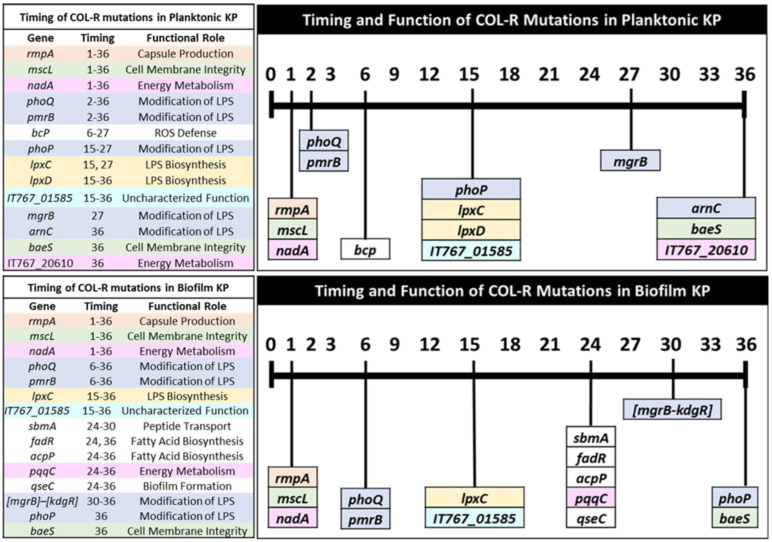
Timing of colistin-resistant mutations by functional role for independent KP lifestyles. Mutations are listed in relation to the first appeared time during evolution and color-coded by functional role to compare COL-R resistance patterns in planktonic and biofilm lifestyles. The timing in which shared mutations are generated and their corresponding functions can be compared between planktonic and biofilm-evolved KP. The colors in the bars are used to identify the individual or combinations of mutations generated at a similar timeframe throughout colistin selection. There is no connection between the color scheme used here and in other figures in this manuscript.

Planktonic and biofilm-evolved KP in this study shared many COL-R mutations with various functions in capsule production, cell membrane integrity, energy metabolism, and modification of LPS structure and biosynthesis (Table 3 and Table 4). Defense against reactive oxygen species (ROS) by *bcp* was a mutation only observed in planktonic-evolved KP, while mutations in genes regulating fatty-acid biosynthesis (*fadR* and *acpP*), biofilm formation (*qseC*), and peptide transport (*sbmA*) were only observed in biofilm-evolved KP (Table 3 and Table 4). A mutation in DUF3413 domain-containing protein was observed in both planktonic and biofilm KP, but remains functionally uncharacterized. However, the generation time of this mutation appears to align with other mutations on genes responsible for the modification of LPS (*lpxC* and *lpxD*) (Figure 4). Next, we assessed the timing of mutations according to their functional roles observed for both planktonic- and biofilm-evolved KP to better understand their influence on the COL-R mechanism.

#### 3.4.1. Capsule Production

Hypervirulent KP has been characterized on the basis of several features, including the virulence gene *rmpA*, a regulator of the mucoid phenotype A, which is an activator for capsular polysaccharide synthesis [30,31]. Low *rmpA* expression has been shown to be correlated with a hypervirulence-negative phenotype in KP [30]. However, less is understood regarding the impact of *rmpA* on COL-R and the potential evolutionary tradeoff between hypervirulence and COL-R. An insertion in *rmpA* was acquired by day 1 of selection in both bacteria lifestyles and was nearly fixed after 24 h. The timing of this insertion aligns with 64-, 32-, and 32-fold increases in MIC for planktonic populations 1–3 (Table 3) and twofold increases in MIC for biofilm populations (Table 4). Notably, these MIC increases were similarly observed for *mscL* and *nadA* mutations, which have functions in cell membrane integrity and energy metabolism, respectively. Our results suggest that capsule production is one of the earliest functional groups modified by colistin selection (Figure 4).

#### 3.4.2. Cell Membrane Integrity

Genes with roles in cell membrane integrity that were altered following colistin selection include *mscL* and *baeS*, which were generated by day 1 and day 36 in planktonic and biofilm populations, respectively (Table 3 and Table 4, Figure 4). SNPs in *baeS* were observed in planktonic population 3 and biofilm population 2 on the final day of selection at 75% and 100% frequency, respectively (Table 3 and Table 4).

#### 3.4.3. Energy Metabolism

Quinolinate synthase NadA, XylR family transcriptional regulator, and pyrroloquinoline–quinone synthase PqqC are associated with energy metabolism and were modified through colistin selection. SNPs in *nadA* were generated by day 1 and persisted throughout selection. Through carbon catabolite repression, bacteria may activate transcription factor XylR to regulate the metabolism of _L_-arabinose and _D_-xylose in place of glucose [32]. Mutations in XylR family transcriptional regulator were only acquired in planktonic populations 2 and 3 on day 36 (Table 3). *PqqC* expression is required for the biosynthesis of pyrroloquinoline quinone, a vitamin and redox cofactor of bacterial dehydrogenases, important for cell growth and metabolic reactions [33]. A mutation in *pqqC* was generated only in biofilm population 3 on day 24 but persisted until the end of selection at 100% frequency (Table 4). While mutations in *nadA* were fixed early in selection, mutations in *xylR* and *pqqC* were required at later timepoints for planktonic and biofilm populations, respectively (Figure 4). It is likely that alterations in bacterial metabolism are direct responses to the environmental stress posed by increasing colistin selection pressure.

#### 3.4.4. Modification of LPS

The disruption of polymyxin interactions with negatively charged phosphate groups of lipid A of LPS is a commonly observed mechanism of COL-R [34]. Several genes involved in the modification of LPS were observed for both lifestyles through selection, including *mgrB*, *phoQ*, *phoP*, and *pmrB*. Two-component transduction systems PmrAB and PhoPQ regulate the modifications of LPS in response to colistin or a decline or rise in Mg^2+^ and Fe^3+^ levels, respectively [35]. Activation of the PhoPQ signaling system leads to the synthesis of small regulatory transmembrane protein MgrB, which functions as a negative feedback regulator of PhoPQ systems [36]. SNPs in *phoQ* were acquired early in planktonic KP, by days 2, 3, and 27 for populations 1–3. A SNP in *pmrB* was acquired by day 2 and persisted until day 27 in population 3, while another SNP in *pmrB* was generated by day 36 in population 1. Mutations in *phoP* were generated by days 27 and 15 for populations 1 and 3. A single SNP in *mgrB* was observed on day 27 for population 1 at 71% frequency (Table 3). *ArnC*, which encodes undecaprenyl-phosphate 4-deoxy-4-formamido-L-arabinose transferase, was also modified in planktonic population 3 by day 36 (81%). ArnC is one of several enzymes involved in adding an amino sugar _L_-Ara4N to lipid A, which disrupts the interaction of cationic peptides with LPS, leading to rapid colistin resistance [37].

For biofilm populations, SNPs in *phoQ* and *pmrB* were generated by day 6 in populations 1 and 3, while a single SNP in *phoP* was generated on day 36 in population 3. Multiple SNPs in *phoQ* were generated in biofilm population 2 at unique positions, with each additional mutation aligning with a substantial rise in MIC (Table 4). A single deletion in *mgrB*–*kdgR* was generated on day 30 and was subsequently fixed. Overall, it appears that mutations involved in LPS modifications are observed early on (by days 2–6) in selection and reappear at later timepoints (days 27–36) for both planktonic and biofilm populations. Additionally, mutations involved in LPS biosynthesis are generated around or shortly following modifications in LPS (Figure 4).

#### 3.4.5. LPS Biosynthesis

In our experimental evolution, we observed mutations in *lpxc* and *lpxD* at later timepoints of selection in both planktonic and biofilm KP (Figure 4). SNPs in *lpxD* were acquired by days 15–27 for all planktonic populations, while SNPs in *lpxC* were observed on days 27 and 15 for populations 2 and 3, corresponding to a high level of COL-R (512 μg/mL) (Table 3). For biofilm populations 1 and 3, SNPs in *lpxC* were observed by day 15 and 24 at 100% frequency.

#### 3.4.6. ROS Defense

Mutations in bacterioferritin comigratory protein (BCP) were solely observed in planktonic populations 1 and 2 through selection. A single mutation in *bcp* was acquired on day 6 that persisted until day 27 in population 1, which aligned with a 128–512-fold increase in MIC from baseline. Two mutations in *bcp* were observed in population 2 on days 15–27 and day 36, consistent with 512–2048-fold increases in MIC from baseline (Table 3). BCP has roles in bacteria’s defense against environmental ROS such as hydrogen peroxide [38]. It is likely that mutations in *bcp* are responses to oxidative stress posed by colistin treatment on bacterial cells.

#### 3.4.7. Peptide Transport

A single mutation in peptide antibiotic transport gene *sbmA* was observed on days 24–30 for biofilm population 3, with a 256–1024-fold increase in MIC (Table 4). SbmA is an inner membrane transporter that facilitates the transport of antimicrobial peptides, especially those that are proline-rich, into the cell [39]. It has recently been observed that mutations in *sbmA* confer resistance to certain peptide conjugates [40]. Biofilm KP mutations in *sbmA* occurred following LPS biosynthesis modifications, in accordance with two mutations having roles in fatty-acid biosynthesis (Figure 4).

#### 3.4.8. Fatty-Acid Biosynthesis

Two mutations were observed in biofilm population 1 with roles in fatty-acid biosynthesis. A mutation in fatty-acid metabolism transcriptional regulator FadR was observed on days 24 and 36, which corresponded to 256- and 1024-fold increases in MIC from baseline. This gene activates fatty-acid synthesis while repressing fatty-acid degradation in response to environmental fatty-acid levels and may influence bacterial cell size [41]. A mutation in acyl carrier protein ACP was generated and fixed after day 24 and aligned with a 246–1024-fold increase in MIC (Table 4). ACP is a highly conserved transport protein for acyl intermediates and is necessary for fatty-acid biosynthesis [42]. Mutations in *fadR* and *acpP* aligned with the timing of a mutation in *pqqC*, which affects energy metabolism, and a mutation in *qseC*, which has roles in biofilm formation (Figure 4).

#### 3.4.9. Biofilm Formation

A single mutation in a two-component system sensor histidine kinase QseC was generated in biofilm population 2 on day 24 and became fixed. This mutation coincided with a 512–2048-fold increase in MIC from baseline (Table 4). *QseC* is an integral gene of the QseBC two-component system of quorum-sensing with roles in modulating biofilm formation and potentially regulating virulence in KP [43].

#### 3.4.10. Mutations with Uncharacterized Function

A mutation in a DUF3413 domain-containing protein was observed following alterations in LPS synthesis genes *lpxC* and *lpxD*, independent of bacterial lifestyle. While the function of this gene is uncharacterized, it was generated by days 15–27 in planktonic and biofilm populations. The mutations in this gene aligned with a 512-fold increase in MIC from baseline for planktonic populations (Table 3). Biofilm population 2 acquired two independent mutations in this gene; one mutation on day 15 corresponded to a 96-fold increase in MIC from baseline, while another mutation that was fixed after day 24 aligned with 512–2048-fold increases in MIC from baseline. A single mutation was observed on day 15 for population 3, with a 128-fold increase in MIC compared to the baseline (Table 4).

### 3.5. Theoretical Pathways of Colistin Resistance in K. pneumoniae

**Figure 5:**
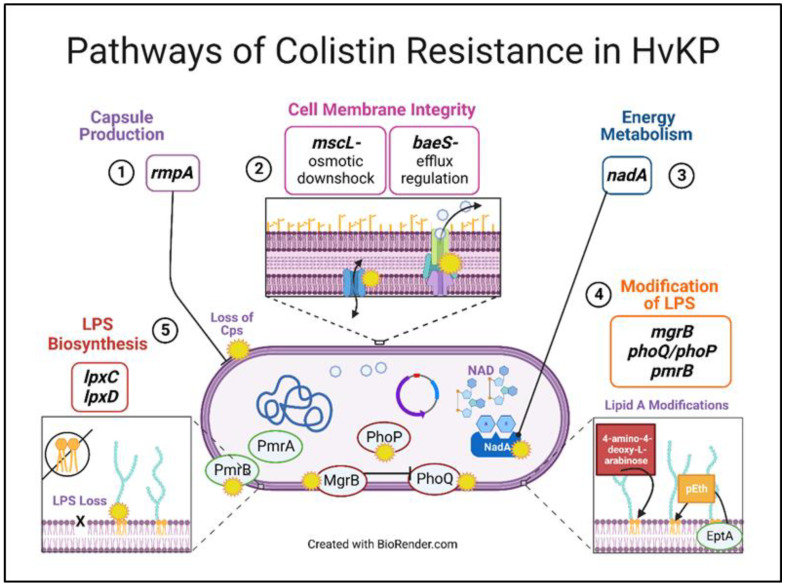
Theoretical pathways of colistin resistance in hypervirulent-*K. pneumoniae*. Colistin selection led to changes in five major gene functional groups, possibly in a sequential manner in both bacterial lifestyles: capsule production, cell membrane integrity, energy metabolism, modifications of LPS, and LPS biosynthesis loss. We posit that the mutations related to these functional groups facilitate and allow for enhanced colistin resistance in *K. pneumoniae*.

On the basis of the similarities in mutations observed with increasing colistin MIC by bacterial lifestyle, we theorize a pathway of colistin resistance, including the timing of affected gene functions and their corresponding mutations (Figure 5). We predict that an initial mutation in *rmpA* leads to loss of capsule polysaccharide (cps) synthesis, followed by regulation in osmotic pressure and efflux (mutations in *mscL* and *baeS*). Next, one could observe changes in energy metabolism through a modification in *nadA*. Additionally, mutations in *mgrB*, *phoQ*/*phoP*, and *pmrB* led to the addition of 4-amino-4-deoxy-L-arabinose and/or the transfer of phosphoethanolamine (pEth) by enzyme phosphoethanolamine transferase. Lastly, mutations in *lpxA* and *lpxD* critical for lipid A synthesis contribute to LPS loss. These mutations allow for the development and progression of colistin resistance in hypervirulent *K. pneumoniae*.

### 3.6. COL-R Isolates Remain Susceptible to Dual-Inhibitor Antibiotics

**Figure 6:**
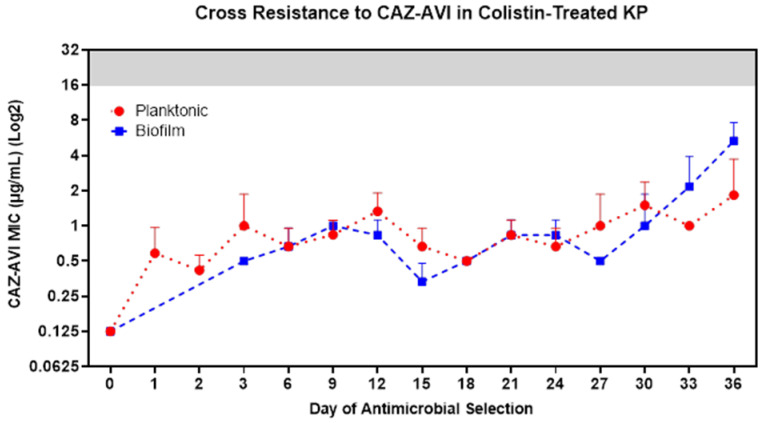
COL-R isolates do not generate cross-resistance to CAZ/AVI. Following 36 days of experimental evolutions of KP ATCC 43816 under colistin selection, cross-resistance to CAZ/AVI was assessed for COL-R planktonic (red) and biofilm (blue) isolates from all populations in all timepoints. The clinical breakpoint for CAZ/AVI is 16 µg/mL, and the resistance MIC area is shown in a gray background. The data points reflect the mean MIC to CAZ/AVI of three replicate populations by lifestyles and colistin treatment conditions. Data are representative of three independent experiments.

Recently, physicians have started using the newly approved β-lactam/β-lactamase dual-inhibitor antibiotics to treat clinical infections that are resistant to colistin treatment. We investigated the potential for cross-resistance of these COL-R isolates to a novel dual-inhibitor antibiotic, ceftazidime–avibactam (CAZ/AVI). Throughout the 36 day experimental evolution under colistin selection, we also tested the MIC of CAZ/AVI for both planktonic and biofilm populations every three days. We found that both planktonic and biofilm lifestyles showed sustained susceptibility to CAZ/AVI, throughout the 36 days of selection, even with substantial COL-R progression (Figure 6).

### 3.7. Loss of Hypermucoviscous Phenotype with Colistin Selection

Our results indicated that rapid COL-R was initially dependent on mutations in mucoid phenotype A regulator gene *rmpA*, leading to changes in capsular polysaccharide synthesis and the hypermucoviscous (HMV) phenotype, independent of bacterial lifestyle. To demonstrate the *rmpA* mutations and alterations in HMV, we conducted the string test for hypervirulence on 1 day evolved (under ½ MIC colistin treatment) planktonic population clones compared to the evolutionary ancestor clone. We found a significant decrease in the percentage of colonies that passed string tests following 1 day of colistin treatment for all three planktonic populations (Supplementary Figure S2). These results suggest that there may be an evolutionary tradeoff between hypervirulence and progressive resistance to colistin.

## 4. Discussion

Colistin resistance has been extensively studied with meaningful mutations and their roles in the resistance mechanism identified. Modifications in lipid A moiety of LPS, overexpression of two-component regulatory systems PhoPQ and PmrAB, plasmid-mediated transfer of mobilized colistin resistance genes *mcr-1* to *mcr-8*, and the inactivation of the PhoQ/PhoP signaling regulator MgrB are some of the most understood mechanisms of colistin resistance in Gram-negative bacteria [14,37,44,45,46,47,48,49,50]. In this study, we narrow the knowledge gap in understanding the importance of mutation timing in the progression of COL-R by gene function for both planktonic and biofilm KP lifestyles. We show a similar pattern of resistance mutation timing through colistin selection between bacteria lifestyles (Figure 4). The “first wave” of early resistance was consistent between lifestyles, with the generation of mutations relevant to capsule production, cell membrane integrity, and energy metabolism. Mutations in *mscL* and *nadA*, regulating osmotic stress and energy metabolism, respectively, were likely immediate responses to environmental stress posed by colistin treatment. NadA is a catalyst in the biosynthesis of nicotinamide adenine dinucleotide (NAD^+^), an essential cofactor, signaling molecule, and coenzyme for redox reactions of energy metabolism [51,52]. MscL is a pore-forming membrane protein that protects the cell from osmotic downshock, by promoting the efflux of various molecules such as potassium, glutamate, and proline, from the cytosol [53]. MscL is considered a potential drug target by acting as an emergency release valve, allowing for the uptake of extracellular molecules, including antibiotics [54]. These mutations may promote the development of further resistance mutations with roles in LPS modification and synthesis.

Following the “first wave”, sporadic mutations affecting LPS structure were observed after day 2 in planktonic populations and occurred later on, by day 6, in biofilm populations. It appears that the positioning of the two-component system sensor and response regulator mutations responsible for LPS modifications may impact COL-R progression. For instance, for planktonic and biofilm populations, mutations at unique gene positions were generated in *phoQ* and *pmrB*. It is possible that mutation positions facilitating optimized COL-R became fixed in each population through enhanced selection pressure. Interestingly, mutations in LPS biosynthesis, likely leading to LPS loss, were generated following modifications in lipid A. For both lifestyles, lipid A modifications preceded mutations in *lpxC* and *lpxD* genes responsible for LPS biosynthesis. This suggests that LPS production may have been altered through colistin selection. Complete loss of LPS is a known mechanism of COL-R in *A. baumannii*, through modifications in *lpxA*, *lpxC*, and *lpxD*, the primary genes involved in lipid A synthesis, leading to a dramatic increase in colistin MIC of greater than 256 µg/mL [49]. Loss of LPS through deletions in *lpx* genes associated with COL-R has also been observed in *Escherichia coli* [55]. However, less is understood regarding the potential loss of LPS in KP, leading to rapid COL-R. Decreased LPS and modifications in LPS are likely the dominant mechanisms of COL-R observed in both planktonic and biofilm evolutions in our study. In addition, an uncharacterized gene mutation (*IT767_0158*) was detected in both lifestyles following mutations *lpxC* and/or *lpxD*, suggesting that this gene may have roles in regulating LPS. Further investigation is necessary to understand the role of these gene mutations and whether their function is related to LPS biosynthesis.

We also found that bacterial evolution to resistance is likely specialized according to bacterial lifestyle in terms of mutation timing and MIC increase. The biofilm environment itself is designed to protect and respond in defense to environmental stressors, shown by a lag time in COL-R development compared with planktonic-evolved populations. In addition, biofilm development requires more time and metabolic demand for cell attachment, colonization, and maturation, compared to free-living cell propagation. Several mutations were theoretically specialized for biofilm-evolved COL-R, including mutations with roles in peptide transport, fatty-acid biosynthesis, and biofilm formation (*sbmA*, *fadR*, *acpP*, and *qseC*). The sensory kinase QseC is part of the two-component-based quorum sensing system (QseBC), which responds to environmental stress, including changes in osmotic pressure, heat shock, and oxidative stress [56]. Further investigation is required to understand the role of the quorum sensing system QseBC in COL-R development. Mutations generated at later timepoints (on or past day 27) had roles in regulating LPS modifications (*mgrB*, *phoP*, and *arnC*), as well as the envelope stress response and efflux pump expression (*baeS*). Mutations in *baeS*, generated by day 36 for both lifestyles, were likely attributed to bacterial defense against interactions of colistin at the bacterial cell membrane. The membrane-bound sensor histidine kinase BaeS is part of a two-component system involved in envelope stress response that responds to environmental stressors and regulates the expression of different efflux pumps [57]. The pattern of COL-R mutation timing between bacteria lifestyles appears to be both similar and specialized due to growing metabolic demands and likely differences in bacteria susceptibility to colistin treatment.

Through our long-term 36 day experimental evolution, we were able to monitor changes in mutation position and frequency and addition of newly acquired mutations, with the intent to better understand how KP continues to adapt to increasing colistin pressure. These methods allowed us to assess the temporal regulation and interactions of COL-R mutations for over 374 generations (36 days × ~10.2 generations per day). Additionally, we were able to study the timing of various impacted COL-R genes and their functions by bacterial lifestyle. By performing the experimental evolutions using both planktonic and biofilm growth, we were able to account for the effects and demands of bacteria lifestyle as a part of the COL-R mechanism and compare/contrast the most beneficial mutations that led to substantial colistin MIC increase with enhanced selection pressure.

A limitation of in vitro experimental evolution studies is the inability to capture the resistance mechanisms associated with plasmid-mediated transfer in addition to somatic gene mutations. As expected, we did not observe any horizontal transfer of plasmid-borne COL-R genes such as the *mcr* variants, which have been rising in prevalence in nature and human patients [15]. Nonetheless, the system of in vitro experimental evolutions under a controlled environment allows us to unmistakably identify the most beneficial chromosomal alterations leading to progressively enhanced colistin resistance.

Considering the rapid resistance to colistin detected for KP in both lifestyles, it is crucial to determine if the COL-R isolates that could exist in clinical settings will remain susceptible to the newly approved β-lactam/β-lactamase dual-inhibitor antibiotics. CAZ/AVI has been approved for administration in the United States since 2015 to combat ESBL and carbapenemase-producing infections. Avibactam prevents ceftazidime hydrolysis by carbapenemases (KPCs) and ESBLs, while the third-generation cephalosporin ceftazidime exhibits bactericidal activity by inhibiting cell-wall synthesis [58]. Our results demonstrate the diversity in bacterial resistance mechanisms to various antimicrobials, including AMPs and conventional dual-inhibitor antibiotic treatments designed to prevent resistance development. These results support that CAZ/AVI remains a critical treatment option for colistin-resistant infections, which is increasingly vital with the rise in high mortality and hypervirulent MDR bacterial infections in the clinical setting.

In summary, this study presents a clearer understanding of the timing and significance of COL-R mutations in KP to ultimately aid in the development of new clinical treatments that successfully eradicate and/or prevent CR-KP infections in patients. Experimental approaches using in vitro and in vivo models of antibiotic selection similar to laboratory systems described here and isolates sampled from patients before and after treatment [59,60] would facilitate the identification of potentially critical resistance mutations. In addition, we can study the influence of epigenetic factors such as DNA methylation of bacteria, which regulate the expression of genes [61]. With further gain- or loss-of-function studies to investigate the impact of individual and combinations of mutations on antimicrobial resistance [62,63,64], these data are useful for the clinical situation by elucidating the critical targets that are utilized by bacteria for adaptive resistance to antimicrobials. Along with understanding the influence of timing of critical mutations and timing of affected genes by their functional roles for cell survival through adaptive evolution to resistance, studies like this will be important in enhancing drug development to prevent or lessen the rapid resistance to antimicrobials.

## Figures and Tables

**Table 1:** Timepoint and MIC of acquired COL-R mutations in planktonic evolved KP populations. COL-R mutations are represented for each individually evolved KP population with colistin MIC (µg/mL) and population frequency. Mutations with shared gene positions between populations are shaded gray. Shared mutations in genes that are acquired in independently evolved populations are color-coded for comparison. Mutations unique to individual populations are in white background. The shading is darker for mutations with higher population frequency.

**Population 1**	**Position**	**Mutation**	**Gene**	**Day 1: 64**	**Day 2: 64**	**Day 3: 64**	**Day 6: 128**	**Day 15: 512**	**Day 27: 512**	**Day 36: 2048**
	796,797	SNP	*mscL*	94.20%	100%	100%	100%	100%	100%	100%
	2,898,145	SNP	*nadA*	94.50%	100%	100%	100%	100%	100%	100%
	4,782,985:1	INS	*rmpA*	100%	100%	100%	100%	100%	100%	100%
	3,366,007	SNP	*phoQ*		26.30%	9.80%	92.50%	100%	100%	
	5,228,037	SNP	*bcp*				24.80%	100%	100%	
	2,287,052	SNP	*lpxD*					15.00%	100%	
	4,585,314	SNP	*mgrB*						70.80%	
	3,366,111	SNP	*phoQ*							100%
	2,949,548	SNP	*pmrB*							68.60%
	363,465	SNP	*mdtO*	12.20%						
**Population 2**	**Position**	**Mutation**	**Gene**	**Day 1: 32**	**Day 2: 256**	**Day 3: 512**	**Day 6: 512**	**Day 15: 512**	**Day 27: 512**	**Day 36: 2048**
	796,797	SNP	*mscL*	94.20%	100%	100%	100%	100%	100%	100%
	2,898,145	SNP	*nadA*	94.50%	100%	100%	100%	100%	100%	100%
	4,782,985:1	INS	*rmpA*	100%	100%	100%	100%	100%	100%	100%
	3,367,207	SNP	*phoQ*			12.60%	79.30%	100%	100%	
	3,366,550	SNP	*phoQ*			25.10%	14.70%			
	5,228,086	SNP	*bcp*					85.00%	85.50%	
	2,287,052	SNP	*lpxD*						10.40%	
	2,287,023	SNP	*lpxD*							100%
	3,366,222	SNP	*phoQ*							100%
	5,228,407	DEL	*bcp*							100%
**Population 3**	**Position**	**Mutation**	**Gene**	**Day 1: 32**	**Day 2: 64**	**Day 3: 64**	**Day 6: 64**	**Day 15: 512**	**Day 27: 512**	**Day 36: 2048**
	796,797	SNP	*mscL*	94.20%	100%	100%	100%	100%	100%	100%
	2,898,145	SNP	*nadA*	94.50%	100%	100%	100%	100%	100%	100%
	4,782,985:1	INS	*rmpA*	100%	100%	100%	100%	100%	100%	100%
	2,949,368	SNP	*pmrB*				12.60%	86.30%	63.60%	
	3,367,778	SNP	*phoP*						33.00%	
	3,366,214	SNP	*phoQ*						50.30%	
	2,287,022	SNP	*lpxD*						37.60%	
	3,367,218	SNP	*phoQ*						22.40%	
	3,366,222	SNP	*phoQ*							72.90%
	796,974	SNP	*mscL*							21.00%
	3,366,013	SNP	*phoQ*							15.90%

Abbreviations: single-nucleotide polymorphism—SNP; insertion—INS; deletion—DEL.

**Table 2:** Timepoint and MIC of acquired COL-R mutations in biofilm evolved KP populations. COL-R mutations are represented for each individually evolved KP population with colistin MIC (µg/mL) and population frequency. Mutations with shared gene positions between populations are shaded gray. Shared mutations in genes that are acquired in independently evolved populations are color-coded for comparison. Mutations unique to individual populations are in white background. The shading is darker for mutations with higher population frequency.

**Population 1**	**Position**	**Mutation**	**Gene**	**Day 1: 2**	**Day 2: 8**	**Day 3: 128**	**Day 6: 128**	**Day 15: 256**	**Day 24: 256**	**Day 30: 512**	**Day 36: 1024**
	796,797	SNP	*mscL*	100%	100%	100%	100%	100%	100%	100%	100%
	2,898,145	SNP	*nadA*	100%	100%	100%	100%	100%	100%	100%	100%
	4,782,985:1	INS	*rmpA*	100%	100%	100%	100%	100%	100%	100%	100%
	2,949,706	SNP	*pmrB*				47.60%				
	2,949,547	SNP	*pmrB*				40.70%				
	2,949,548	SNP	*pmrB*				38.60%				
	2,950,483	SNP	*pmrA*				36.40%				
	3,367,219	SNP	*phoQ*				13.10%		52.20%		
	2,178,392	SNP	*lpxC*					100%			
	2,178,666	SNP	*lpxC*						36.70%		
	4,585,299	DEL	*[mgrB]*–*[kdgR]*							100%	100%
	2,950,399	SNP	*pmrA*								100%
**Population 2**	**Position**	**Mutation**	**Gene**	**Day 1: 2**	**Day 2: 2**	**Day 3: 16**	**Day 6: 64**	**Day 15: 96**	**Day 24: 512**	**Day 30: 512**	**Day 36: 2048**
	796,797	SNP	*mscL*	100%	100%	100%	100%	100%	100%	100%	100%
	2,898,145	SNP	*nadA*	100%	100%	100%	100%	100%	100%	100%	100%
	4,782,985:1	INS	*rmpA*	100%	100%	100%	100%		100%	100%	100%
	3,366,550	SNP	*phoQ*				74.60%				
	2,949,916	SNP	*pmrB*					75.90%			
	3,366,423	SNP	*phoQ*					33.90%			
	554,800	DEL	*qseC*						100%	100%	100%
	3,365,950	SNP	*phoQ*						100%	100%	100%
	4,903,958	SNP	*baeS*								100%
**Population 3**	**Position**	**Mutation**	**Gene**	**Day 1: 2**	**Day 2: 16**	**Day 3: 64**	**Day 6: 64**	**Day 15: 128**	**Day 24: 256**	**Day 30: 1024**	**Day 36: 2048**
	796,797	SNP	*mscL*	100%	100%	100%	100%	100%	100%	100%	100%
	2,898,145	SNP	*nadA*	100%	100%	100%	100%	100%	100%	100%	100%
	4,782,985:1	INS	*rmpA*	100%	100%	100%	100%	100%	100%	100%	100%
	864,664	SNP	*envZ*					100%			
	2,178,740	SNP	*lpxC*						100%	100%	100%
	2,949,703	SNP	*pmrB*						100%	100%	100%
	864,325	SNP	*envZ*								61.80%
	3,367,440	SNP	*phoP*								45.40%

Abbreviations: single-nucleotide polymorphism—SNP; insertion—INS; deletion—DEL.

**Table 3:** Functional roles of COL-R mutations in relation to timing, population frequency (percentage), and colistin MIC (µg/mL) for planktonic-evolved KP. Resistant MICs are shown in red, and mutations independent to planktonic lifestyle are shown in blue.

Planktonic KP Colistin-Resistance Mutations by Functional Role and MIC											
			Population 1			Population 2			Population 3		
**Gene**	Description	Function	Day	Freq %	MIC µg/mL	Day	Freq %	MIC µg/mL	Day	Freq %	MIC µg/mL
*rmpA*	mucoid phenotype A regulator	Capsule production	1–36	100	64–2048	1–36	100	32–2048	1–36	100	32–2048
*mscL*	large-conductance mechanosensitive channel protein	Cell Membrane Integrity	1–36	94–100	64–2048	1–36	92–100	32–2048	1–36	100	32–2048
*baeS*	two-component system sensor histidine kinase		-	-	-	-	-	-	36	75	2048
*nadA*	quinolinate synthase	Energy Metabolism	1–36	95–100	64–2048	1–36	91–100	32–2048	1–36	95–100	32–2048
*IT767_20610*	XylR family transcriptional regulator		-	-	-	36	100	2048	36	83	2048
*mgrB*	PhoP/PhoQ regulator	Modification of LPS	27	71	512	-	-	-	-	-	-
*phoQ*	two-component system sensor histidine kinase		2–27 36	26–100	64-2048	3–6, 3–27 36	13–100	512–2048	27 36	22 73	512 2048
*phoP*	two-component system response regulator		27	10–15	512	-	-	-	15–27	8–33	512
*pmrB*	two-component system sensor histidine kinase		36	69	2048	-	-	-	2–27	7–86	64–512
*arnC*	undecaprenyl-phosphate 4-deoxy-4-formamido-L-arabinose transferase		-	-	-	-	-	-	36	81	2048
*lpxD*	UDP-3-O-(3-hydroxymyristoyl)glucosamine N-acyltransferase	LPS biosynthesis	15–27	15–100	512–2048	27 36	10 100	512 2048	27	38	512
*lpxC*	UDP-3-O-acyl-N-acetylglucosamine deacetylase		-	-	-	27	22	512	15	14	512
*IT767_01585*	DUF3413 domain-containing protein	Unknown	15	16	512	27	30	512	27–36	100	512–2048
*bcp*	thioredoxin-dependent thiol peroxidase	ROS Defense	6–27	25–100	128–512	15–27 36	8–100	512–2048	-	-	-

Abbreviations: mutation frequency—Freq; hypermucoviscous—HMV; nicotinamide adenine dinucleotide—NAD; lipopolysaccharide—LPS; reactive oxygen species—ROS.

**Table 4:** Functional roles of COL-R mutations in relation to timing, population frequency (percentage), and colistin MIC (µg/mL) for biofilm-evolved KP. Resistant MICs are shown in red, sensitive MICs are shown in green, and mutations independent to biofilm lifestyle are shown in green.

Biofilm KP Colistin-Resistance Mutations by Functional Role and MIC											
			Population 1			Population 2			Population 3		
Gene	Description	Function	Day	Freq %	MIC µg/mL	Day	Freq %	MIC µg/mL	Day	Freq %	MIC µg/mL
*rmpA*	mucoid phenotype A regulator	Capsule production	1–36	100	2– 1024	1–36	100	2– 2048	1–36	100	2– 2048
*mscL*	large-conductance mechanosensitive channel protein	Cell Membrane Integrity	1–36	100	2– 1024	1–36	100	2– 2048	1–36	100	2– 2048
*baeS*	two-component system sensor histidine kinase		-	-	-	36	100	2048	-	-	-
*nadA*	quinolinate synthase	Energy Metabolism	1–36	100	2– 1024	1–36	100	2– 2048	1–36	100	2– 2048
*pqqC*	pyrroloquinoline-quinone synthase		-	-	-	-	-	-	24–36	100	256–2048
*[mgrB]*–*[kdgR]*	PhoP/PhoQ regulator - DNA-binding transcriptional repressor	Modification of LPS	30–36	100	512–1024	-	-	-	-	-	-
*phoQ*	two-component system sensor histidine kinase		6, 24	13, 52	128, 256	6, 15, 24–36	34–100	64–2048	-	-	-
*phoP*	two-component system response regulator		-	-	-	-	-	-	36	45	2048
*pmrB*	two-component system sensor histidine kinase		6, 6, 6	39–48	128	15	76	96	24–36	100	256–2048
*lpxC*	UDP-3-O-acyl-N-acetylglucosamine deacetylase	LPS Biosynthesis	15, 24	100, 37	256	-	-	-	24–36	100	256–2048
*IT767_01585*	DUF3413 domain-containing protein	Unknown	-	-	-	15, 24–36	63, 100	96, 512–2048	15	94	128
*fadR*	fatty acid metabolism transcriptional regulator	Fatty Acid Biosynthesis	24, 36	34, 100	256, 1024	-	-	-	-	-	-
*acpP*	acyl carrier protein		24–36	46–100	256–1024	-	-	-	-	-	-
*qseC*	two-component system sensor histidine kinase	Biofilm Formation	-	-	-	24–36	100	512–2048	-	-	-
*sbmA*	peptide antibiotic transporter	Peptide Transport	-	-	-	-	-	-	24–30	52–70	256–1024

Abbreviations: mutation frequency—Freq; hypermucoviscous—HMV; nicotinamide adenine dinucleotide—NAD; lipopolysaccharide—LPS; antimicrobial peptide—AMP.

## Data Availability

Data are contained within the article or Supplementary Materials. The sequencing data have been deposited with links to BioProject accession number PRJNA922161 in the NCBI BioProject database (https://www.ncbi.nlm.nih.gov/bioproject/PRJNA922161).

## Supplementary Materials

The following supporting information can be downloaded at https://www.mdpi.com/article/10.3390/pharmaceutics15010270/s1: Figure S1. Schematic of planktonic and biofilm experimental evolution workflow; Figure S2. Evolved KP isolates quickly lost their hypervirulence.
