# Supplementary material for: Determination of Mutational Timing of Colistin-Resistance Genes through Klebsiella pneumoniae Evolution
Source: Pharmaceutics. 2023 Jan 12;15(1):270. doi: 10.3390/pharmaceutics15010270 (PMC9862994; doi:10.3390/pharmaceutics15010270)
Supplement: Supplementary file 1 [file pharmaceutics-15-00270-s001.zip › pharmaceutics-2068679-SI.pdf]

Supplementary Materials

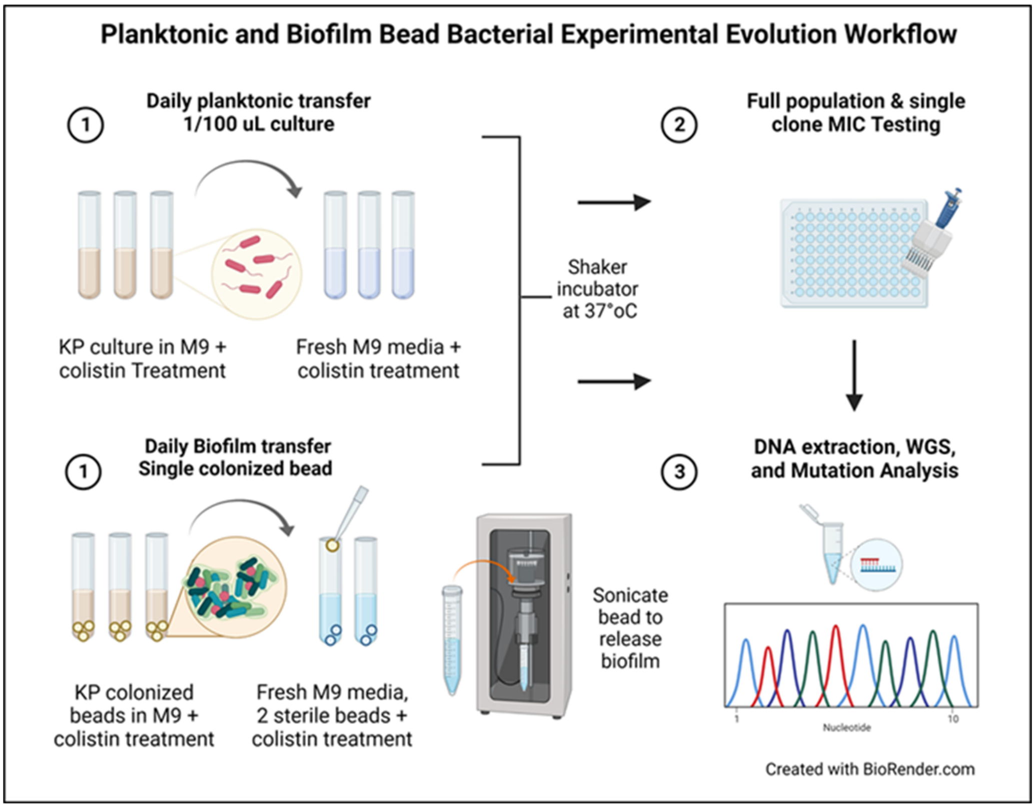

Supplementary Figure S1. Schematic of planktonic and biofilm experimental evolution workflow. Experimental evolutions were performed using KP (ATCC 43816) as an ancestor and growing in planktonic and biofilm bead transfer lifestyles. Three independently evolved populations from each lifestyle under colistin selection were used to compare the resistance mechanisms. Whole population MIC testing was conducted daily for the first three days and every 72 hours afterward. DNA was extracted from selected time points for further genomic analysis to predict mutations computationally. The numbers indicated the stages of experimental approaches.

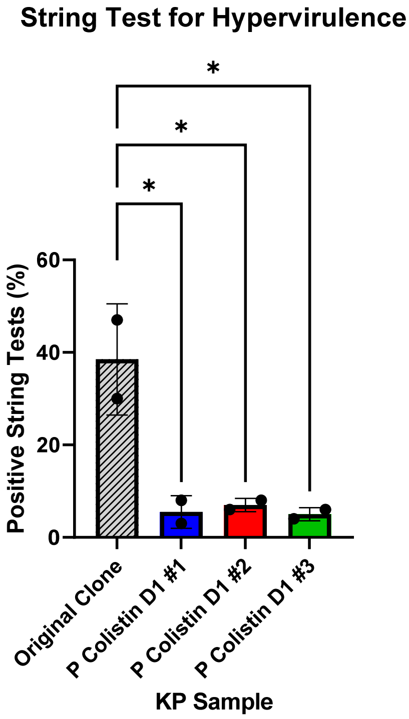

Supplementary Figure S2. Evolved KP isolates quickly lost their hypervirulence. Individual colonies from planktonic colistin-treated populations 1-3 were compared to the original KP ancestor clone (ATCC 43816) for hypervirulence. Single colonies showing a mucoid string of 5mm or greater indicated a positive string test for hypervirulence. Mutations in mucoid phenotype A regulator RmpA potentially lead to loss of hypervirulence as one of the KP colistin resistance mechanisms. . *$p < 0.05$ indicates a significant difference in positive string tests compared to the original clone.
